# Role of Autophagy Inducers and Inhibitors in Intestinal Barrier Injury Induced by Intestinal Ischemia–Reperfusion (I/R)

**DOI:** 10.1155/2022/9822157

**Published:** 2022-07-30

**Authors:** Yuejin Li, Peng Zhang, Jian Zhang, Weimin Bao, Jinyuan Li, Yan Wei, Jinchao Ni, Kunmei Gong

**Affiliations:** Department of General Surgery, The First People's Hospital of Yunnan Province, The Affiliated Hospital of Kunming University of Science and Technology, Kunming, 650032 Yunnan Province, China

## Abstract

**Objectives:**

Intestinal epithelial barrier function is an important mechanical barrier to maintain intestinal homeostasis and resist the invasion of intestinal pathogens and microorganisms. However, intestinal epithelial barrier function is vulnerable to damage under intestinal ischemia–reperfusion (I/R) injury. Under a category of pathophysiological conditions, including I/R, autophagy plays a crucial role. This study is aimed at discussing the role of autophagy inhibitors and activators in intestinal epithelial barrier function after intestinal I/R by changing autophagy levels.

**Methods:**

Mice with intestinal IR underwent 45 minutes of surgery for superior mesenteric artery occlusion. The autophagy inhibitor 3-MA and the autophagy inducer rapamycin (RAP) were used to change the level of autophagy, and then, the expressions of tight junction proteins and intestinal barrier function were detected.

**Results:**

The results showed that the autophagy inhibitor 3-MA aggravated intestinal epithelial barrier dysfunction, while the autophagy inducer RAP attenuated intestinal epithelial barrier dysfunction. In addition, promoting autophagy may promote occludin expression by inhibiting claudin-2 expression.

**Conclusion:**

Upregulation of autophagy levels by autophagy inducers can enhance intestinal epithelial barrier function after intestinal I/R.

## 1. Introduction

The intestine is an important tissue that transports nutrients for the body in animals and also an important barrier in the body's defense response, which is related to the intestinal mucosal barrier comprising intestinal epithelial cells [1]. The intestinal mucosal barrier is an important part of maintaining the ecological stability of the body. The normal intestinal mucosal barrier can block the invasion of external microorganisms and ensure the balance of microorganisms such that all parts of the tissue can perform normal physiological functions. Once the intestinal mucosal barrier is damaged, the intestinal epithelium is damaged. The permeability of the tissue will change, the bacteria will be ectopic, and the dynamic balance of the microorganisms will be broken, which will lead to the occurrence of an inflammatory response, in severe cases causing life-threatening damage to the surrounding organs and leading to systemic multiple organ dysfunction [2].

Intestinal barrier damage is a common clinical inflammatory disease, and some mechanical damage and drug factors may cause intestinal barrier dysfunction. Ischemia–reperfusion (I/R) is a common clinical condition. A typical complication is intestinal mucosal barrier dysfunction. When physiological conditions (such as exercise and stress) and pathophysiological events (such as acute mesenteric ischemia and aortic surgery) occur, body I/R occurs, followed by an acute cascade of processes, including endothelial destruction, epithelial barrier condensation, immune cell activation, and release of inflammatory mediators [3]. Second, changes in the inflammatory response, oxidative stress, and autophagy mechanisms are also the main reasons for the dysfunction of the intestinal mucosal barrier [4–6]. These reasons are usually mixed, and more in-depth and detailed research is needed as to which dominates and which takes precedence.

Autophagy is an intracellular degradation process that relies on the degradation pathway of lysosomes to maintain cellular homeostasis and participate in tissue ecological regulation, immune response, and antibacterial protection. Autophagy selectively targets dysfunctional organelles. Intracellular microorganisms and pathogenic proteins prevent the occurrence of diseases [7]. There is evidence that autophagy regulates the occurrence of various diseases, including cancer, neurodegenerative diseases, and inflammatory diseases [8–10]. As a common inflammatory disease, intestinal mucosal barrier dysfunction is closely related to autophagy. The normal autophagy mechanism can remove ectopic bacteria caused by changes in intestinal mucosal permeability in a timely manner and maintain the balance of intestinal microbes. The abnormal autophagy mechanism will aggravate the dysfunction of the intestinal mucosal barrier, thereby aggravating the damage to the body. Studies have confirmed the possible role of autophagy in the intestinal mucosal barrier dysfunction caused by I/R and other reasons, and the corresponding autophagy recovery treatment has also been taken as an opportunity and achieved good results. In one study, impaired autophagy was found to be associated with intestinal mucosal dysfunction in the mucosa of mice with colitis, and restoration of the autophagy mechanism after resveratrol treatment alleviated intestinal mucosal barrier dysfunction [11]. Li et al. found that in the case of intestinal ischemia and reperfusion, autophagy regulates barrier dysfunction of the intestinal epithelium [12–15]. In addition, autophagy could enhance intestinal epithelial tight junction (TJ) and epithelial barrier function by targeting claudin-2 protein degradation [16, 17]. However, the role of autophagy activators and inhibitors in epithelial barrier injury and repair after intestinal I/R is unknown.

In this study, intestinal IR mice underwent 45 minutes of surgery for superior mesenteric artery occlusion. The autophagy inhibitor 3-MA and the autophagy inducer rapamycin (RAP) were used to change the level of autophagy, and then, the expressions of tight junction proteins (TJPs) and intestinal barrier function were detected. The results showed that the autophagy level was downregulated after intestinal I/R and that RAP enhanced epithelial barrier function by promoting the degradation of claudin-2.

## 2. Materials and Methods

### 2.1. Intestinal Ischemia–Reperfusion (I/R) Animal Model

Forty adult male C57BL/6 mice (8 weeks, 18-22 g) were purchased from the Hunan SJA Laboratory Animal Co., Ltd. Mice were given *ad libitum* access to food and water and reared individually with a 12 h light/dark cycle. All animal experiments were approved by the Laboratory Animal Ethics Committee of Kunming University of Science and Technology on February 24, 2021.

Based on previous studies, an animal model of intestinal ischemia I/R was established [12, 15]. In brief, mice were anesthetized with 1% sodium pentobarbital (30 mg/kg). All mice were randomly divided into the sham group, I/R group, I/R+RAP group, and I/R+3-MA group. Mice underwent superior mesenteric artery occlusion for 45 min, except for the sham group, which was then reperfused for 2 h. RAP or 3-MA (2 mg/kg, 1 mL) was injected into the mouse peritoneum 30 min before ischemia. After reperfusion, a blood sample and small intestinal tissue (8 cm long) 5 cm from the return blind valve were collected.

### 2.2. Detection of Intestinal Permeability

The intestinal permeability of the mice was measured according to a previous study [18]. Mice were given fluorescein isothiocyanate- (FITC-) dextran from the rectum after 2 h of reperfusion in the conscious state. Blood samples were collected from retroorbital hemorrhage after 4 h and centrifuged at 850 g for 20 min. The standard curve measuring FITC was used.

### 2.3. Quantitative Reverse Transcription Polymerase Chain Reaction (RT–qPCR)

Total RNA was extracted from intestinal segments using TRIzol reagent (Invitrogen, Carlsbad, CA). RT–qPCR analysis was performed on an ABI 7500 real-time PCR system. The relative expression level of mRNA was calculated by the 2^−ΔΔCq^ method [19], and *β*-actin was used to normalize the mRNA level. The primer sequences used were as follows: mouse claudin-2, forward: 5′-GCAAACAGGCTCCGAAGATACT-3′, reverse: 5′-GAGATGATGCCCAAGTACAGAG-3′; mouse *β*-actin, forward: 5′-TGTTACCAACTGGGACGACA-3′, reverse: 5′-CTGGGTCATCTTTTCACGGT-3′.

### 2.4. Western Blotting

The total proteins of intestinal tissues were extracted by radioimmunoprecipitation assay protein lysis buffer (Invitrogen; Thermo Fisher Scientific, Inc., USA). A bisquinolinecarboxylic acid protein kit was used to measure the concentration of proteins. Then, 40 *μ*g of protein from each sample was separated by 10% sodium salt-polyacrylamide gel electrophoresis and transferred to a polyvinylidene fluoride membrane by the electric transfer method. Five percent skimmed milk powder was used blocking for 1 h. After incubation with primary and secondary antibodies, the protein bands were visualized by enhanced chemiluminescence. The primary antibodies were diluted at 1 : 1000 as follows: anti-LC3B (ab48394; Abcam, UK), anti-beclin 1 (ab210498; Abcam, UK), anti-p62 (ab109012; Abcam, UK), anti-ZO-1 (ab96587; Abcam, UK), anti-occludin (ab216327; Abcam, UK), anti-claudin-1 (ab180158; Abcam, UK), anti-claudin-2 (ab53032; Abcam, UK), anti-claudin-3 (ab52231; Abcam, UK), anti-Bcl-2 (ab182858; Abcam, UK), anti-Bax (ab32503; Abcam, UK), anti-cleaved Caspase-3 (ab49822; Abcam, UK), and anti-*β*-actin (ab8226; Abcam, UK). The grayscale value was analyzed by ImageJ.

### 2.5. Hematoxylin-Eosin (H&E) and Immunohistochemistry (IHC) Staining

Intestinal tissue samples were fixed in 4% paraformaldehyde for 24 h and then dehydrated by washing with a series of ethanol. Subsequently, the samples were embedded in paraffin wax and cut into 5 *μ*m sections. The H&E staining procedure was performed according to the manufacturer's instructions. For IHC analysis, the slices were immersed in 3% hydrogen peroxide solution and incubated with primary antibody against LC3 (Abcam; 1 : 200). Then, immunoglobulin and streptavidin combined with horseradish peroxidase were added. Subsequently, the intestinal sections were stained with 3,3′-diaminobenzidine (DAB) and hematoxylin. Both HE staining and IHC staining were performed according to the method of Su and Tang [20].

### 2.6. TdT-Mediated dUTP Nick-End Labeling (TUNEL) Activity Assays

The paraffin-embedded intestine samples were cut into 5 *μ*m slides. Dehydration was performed in gradient-reduced ethanol after dewaxing in xylene. Epithelial cell apoptosis was detected by an apoptosis detection kit (Roche, Switzerland). The nuclei were stained with DAPI (Beyotime, China).

### 2.7. Immunofluorescence Staining

Intestinal segments were fixed in 4% paraformaldehyde and embedded in paraffin. Then, 5 *μ*m slides were made and stained with anti-claudin-2 and anti-occludin according to a previous report [21]. Tissues were incubated with primary antibodies for 60 min and then incubated with diluted 1 : 500 Alexa Fluor IgG for 45 min in blocking solution. Images were obtained with Zeiss microscopes.

### 2.8. Statistical Analysis

The data are shown as the mean ± standard deviation. The comparison of two groups or between multiple groups was performed by *t* test or one-way ANOVA, respectively. The data in this study were normally distributed and were analyzed by variance analysis and a post hoc test (Bonferroni or Tukey's). GraphPad Prism 7 plotted the experimental data. *P* < 0.05 indicates a significant difference. Western blotting and RT–qPCR experiments were performed in 3 technical replicates.

## 3. Results

### 3.1. Autophagy Was Impaired after Intestinal IRI

**Figure 1:**
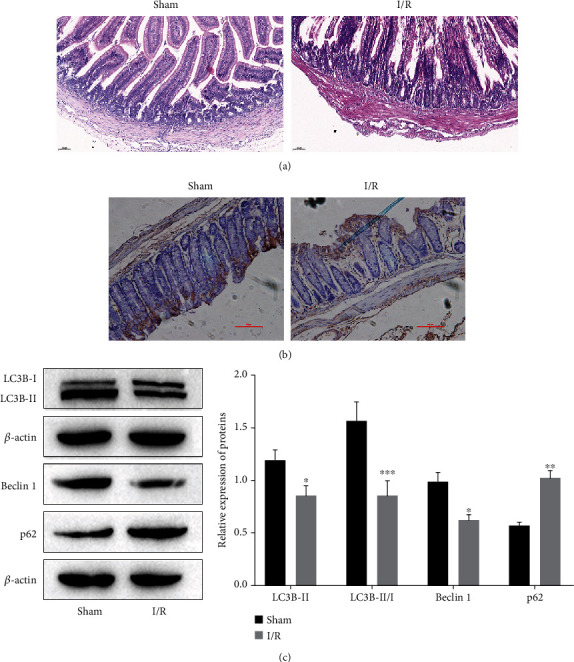
The change in autophagy levels after intestinal IRI. (a) Representative images of H&E staining of intestinal tissues after 2 h of reperfusion. (b) The expression level of LC3B in intestinal tissues was measured by immunohistochemical staining. (c) The expression levels of proteins related to autophagy (LC3B, Beclin 1, and p62) were detected by Western blotting. ^∗^*P* < 0.05, ^∗∗^*P* < 0.01, and ^∗∗∗^*P* < 0.001 vs. the sham group. *n* = 5 per group.

In this study, an intestinal I/R model was established in mice by 45 min of ischemia and 2 h of reperfusion, and this process had no influence on the survival rate of mice according to the observation (data not shown). To confirm whether autophagy inducers and inhibitors affect the function of the epithelial barrier after intestinal IRI, we first detected the autophagy levels in the sham group and I/R group. There was an obvious downregulation of autophagy in intestinal tissues after IRI. After IRI, intestinal villi were intact in the sham group, but the intestinal epithelial barrier in the I/R group was significantly damaged, followed by a large number of epithelial cells uplifting to the villi side and villi falling off (Figure 1(a)). The expression of the autophagy marker LC3B was also decreased after IRI, as shown by the lower level of LC3B-positive cells in intestinal tissues (Figure 1(b)). Furthermore, the LC3B, beclin 1, and p62 expression results showed a decrease in LC3B-II/I and beclin 1, while the relative level of p62 was increased (Figure 1(c)).

### 3.2. TJP Expression Was Impaired after Intestinal IRI

**Figure 2:**
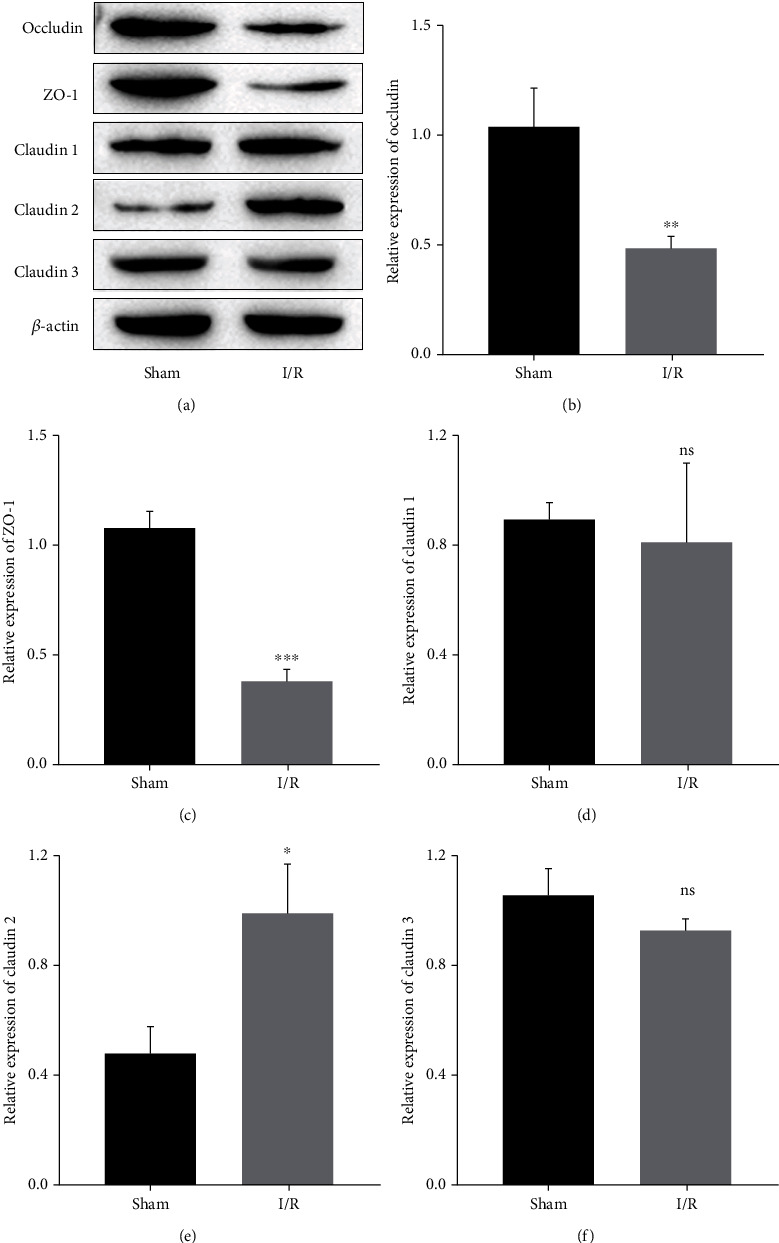
Changes in TJP expression after intestinal IRI. (a) Representative Western blot bands. (b–f) The relative expression levels of occludin, ZO-1, claudin-1, claudin-2, and claudin-3. ^ns^*P* > 0.05, ^∗^*P* < 0.05, ^∗∗^*P* < 0.01, and ^∗∗∗^*P* < 0.001 vs. the sham group. *n* = 5 per group.

To detect dysfunction of the epithelial barrier, the expression of TJPs in intestinal tissues was measured by Western blot assays. The data showed that the expression levels of occludin and zonula occludens protein 1 (ZO-1) were obviously downregulated after intestinal IRI (Figures 2(a)–2(c)). However, for the sham group and I/R group, there was no significant difference between the expression of claudin-1 and claudin-3 (Figures 2(a), 2(d), and 2(f)); meanwhile, compared with the sham group, the expression of claudin-2 in the I/R group was significantly increased (Figures 2(a) and 2(e)).

### 3.3. Effects of RAP and 3-MA on Intestinal Epithelial Cell Apoptosis

**Figure 3:**
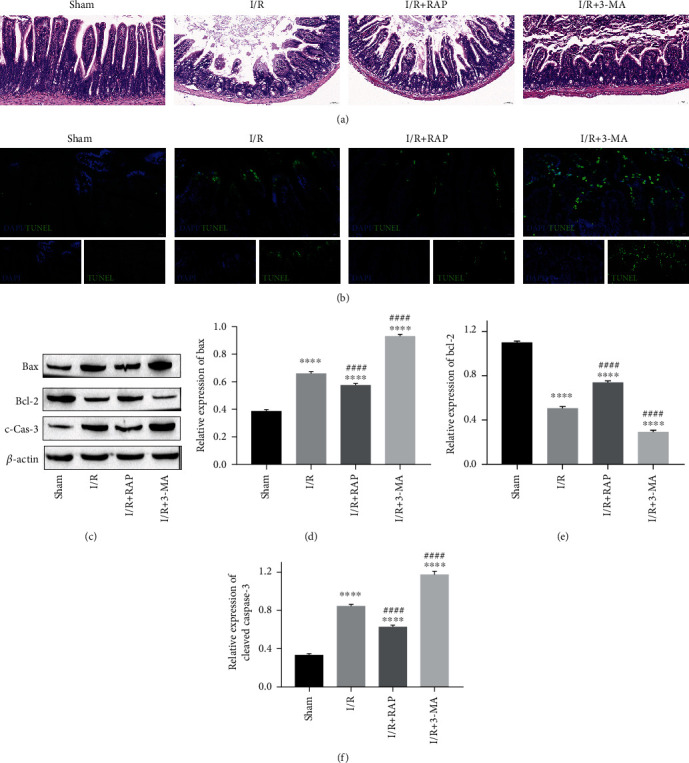
The effects of 3-MA and RAP on intestinal epithelial apoptosis after IRI. (a) Representative images of H&E staining of intestinal tissues in each group. (b) Representative images of intestinal epithelial apoptosis detected by TUNEL activity. (c) The representative bands of Western blotting. (d–f) The expression of Bax, Bcl-2, and cleaved caspase-3. ^∗^*P* < 0.05, ^∗∗∗^*P* < 0.001, and ^∗∗∗∗^*P* < 0.0001 vs. sham group; #*P* < 0.05, and *^####^P* < 0.0001 vs. I/R group. *n* = 5 per group in the sham and I/R groups, and *n* = 10 per group in the other groups.

After confirming the changes in autophagy levels and TJP expression in IRI mice, the effect of the autophagy inducer RAP and the autophagy inhibitor 3-MA on the intestinal epithelial barrier was determined. First, the apoptosis of intestinal epithelial cells was measured. As shown in Figure 3(a), RAP inhibited injury to the epithelial barrier, while 3-MA aggravated the denudation of villi. TUNEL staining showed that the apoptosis rate of epithelial cells was obviously increased in the I/R group and downregulated by RAP treatment but upregulated by 3-MA treatment (Figure 3(b)). Furthermore, the proteins related to apoptosis were measured. The data showed that the expression of proapoptotic proteins was increased in the I/R group. RAP treatment inhibited the expression of Bax and cleaved caspase-3, while 3-MA promoted their expression (Figures 3(c), 3(d), and 3(f)). In contrast, the antiapoptotic protein Bcl-2 was decreased in the I/R group, RAP treatment upregulated its expression, and 3-MA downregulated the expression of Bcl-2 (Figures 3(c) and 3(e)).

### 3.4. Effects of RAP and 3-MA on Autophagy Levels in Intestinal Tissues

**Figure 4:**
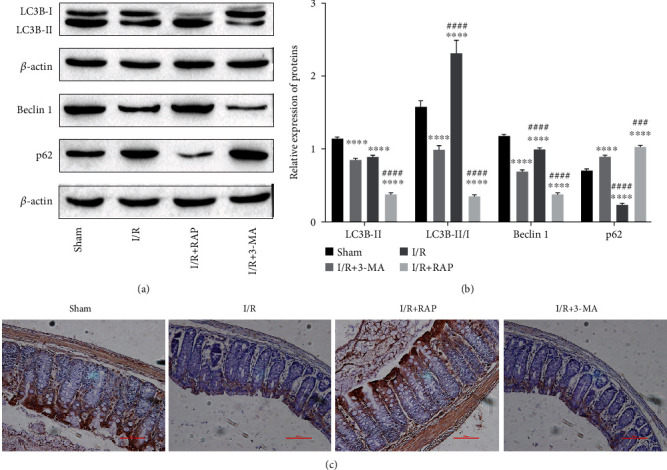
The effects of 3-MA and RAP on autophagy levels in intestinal tissues after IRI. (a) The representative bands of Western blotting. (b) The relative expression levels of LC3B, Beclin 1, and p62. (c) Representative images of immunohistochemical staining for LC3B in each group. ^∗∗∗^*P* < 0.001, ^∗∗∗∗^*P* < 0.0001 vs. the sham group; *^###^P* < 0.001, *^####^P* < 0.0001 vs. the I/R group. *n* = 5 per group in the sham and I/R groups, and *n* = 10 per group in the other groups.

As shown in Figure 4, RAP upregulated the autophagy level, and 3-MA reduced the autophagy level in intestinal tissues after intestinal IRI. Compared with the I/R group, the ratio of LC3B-II/I and beclin 1 was upregulated by RAP treatment but decreased by 3-MA treatment, while the expression of p62 was decreased by RAP and increased by 3-MA (Figures 4(a) and 4(b)). LC3B staining showed an obvious increase in LC3B-positive cells in the RAP-treated group and a downregulation in the 3-MA-treated group compared with the I/R group (Figure 4(c)). These results indicated that RAP and 3-MA could regulate the level of autophagy in intestinal tissues after IRI. However, there were no further data to confirm whether they regulated autophagy levels by directly affecting the autophagy pathway as they are nonspecific autophagy inducers and inhibitors, respectively.

### 3.5. Effects of RAP and 3-MA on the Expression of TJPs

**Figure 5:**
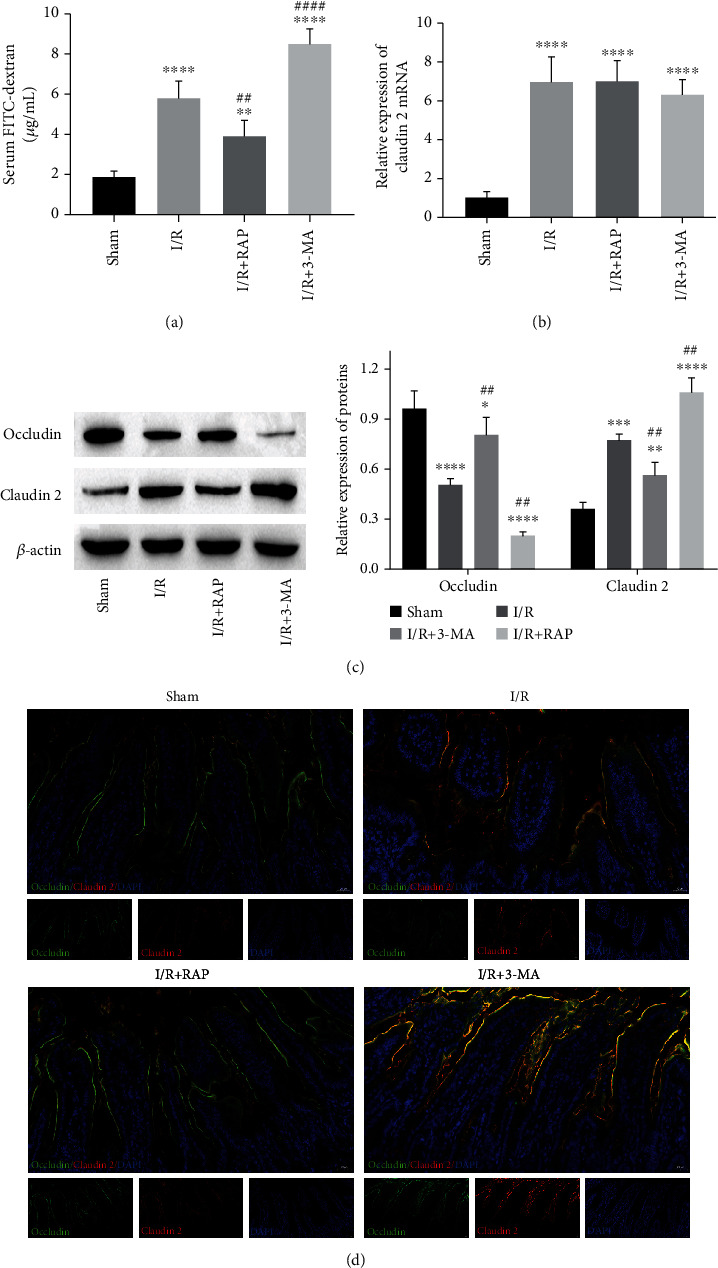
The effects of 3-MA and RAP on the epithelial barrier after intestinal IRI. (a) The change in intestinal permeability was detected by the concentrations of serum FITC-dextran. (b) The relative level of claudin-2 mRNA was measured by RT–qPCR. (c) The protein expression levels of occludin and claudin-2. (d) Representative images of occludin/claudin-2 coimmunofluorescence staining in the intestine after IRI. ^∗^*P* < 0.05, ^∗∗^*P* < 0.01, ^∗∗∗^*P* < 0.001, and ^∗∗∗∗^*P* < 0.0001 vs. the sham group; *^##^P* < 0.01, *^####^P* < 0.0001 vs. the I/R group. *n* = 5 per group in the sham and I/R groups, and *n* = 10 per group in the other groups.

To evaluate the effect of RAP and 3-MA on epithelial barrier function, intestinal permeability and TJP expression were determined. As shown in Figure 5(a), the intestinal permeability of the I/R group was significantly increased compared with that of the sham group. RAP treatment decreased the serum FITC-dextran concentration, and 3-MA increased the concentration of serum FITC-dextran compared with that of the I/R group, indicating that autophagy induction could enhance the function of the intestinal epithelial barrier. The RT–qPCR results showed that IRI induced claudin-2 mRNA expression, but RAP and 3-MA had no influence on claudin-2 mRNA expression (Figure 5(b)).  Figure 5(c) shows that RAP inhibited the protein expression of claudin-2, but 3-MA promoted claudin-2 protein expression compared with I/R; in contrast, the expression of occludin was upregulated by RAP but downregulated by 3-MA. Occludin/claudin-2 coimmunofluorescence staining of intestinal tissues showed that I/R promoted the membrane expression of claudin-2 and its colocalization with occludin, which was aggravated by 3-MA treatment but reversed by RAP treatment (Figure 5(d)).

## 4. Discussion

IRI is considered a severe clinical complication that can cause multiple organ failure and remains clinically challenging in critical patients [22]. With high morbidity and mortality, many factors are involved in the progression of IRI [23]; however, the mechanism of the injury formation process after intestinal IRI and the available treatment for intestinal IRI need to be further studied. Autophagy is a self-renewal and protective mechanism of cells against various stress reactions [24]. Wu et al. found that under a category of pathophysiological conditions, including I/R, autophagy plays a crucial role [6]. Chen et al. revealed that RAP can significantly enhance the protective effect of ischemic postconditioning, while 3-MA weakens the protective effect of ischemic postconditioning [25]. These findings suggest that autophagy plays an important role in intestinal I/R. In this study, we determined the role of the nonspecific autophagy inducer RAP and the nonspecific autophagy inhibitor 3-MA in the injury and repair of the epithelial barrier early after intestinal IRI. Our data showed that RAP-induced autophagy of intestinal epithelial cells enhanced the function of the epithelial barrier, while inhibition of autophagy by 3-MA aggravated epithelial barrier dysfunction early after intestinal IRI. However, another study found results that are contrary to this conclusion, and it confirmed that an induction of autophagy in the first hour of IRI may lead to more damage, while inducing autophagy may be beneficial in the long term for inducing tissue repair [26].

Intestinal epithelial cells regulate the transport of substances and resist the invasion of pathogenic microorganisms by forming a mechanical barrier between the intestinal stromal and the intestinal lumen environment [27, 28]. The survival and death of intestinal epithelial cells are crucial for maintaining a continuous monolayer of epithelium, and accumulating evidence has revealed that autophagy helps to maintain the stability of intestinal epithelium [14, 29]. In this study, we found an upregulation of the apoptotic rate and a downregulation of the autophagic rate in epithelial cells after intestinal IRI, and they were associated with an increase in intestinal permeability and a decrease in the expression of important TJPs (occludin and ZO-1). In addition, the autophagy inhibitor 3-MA promoted epithelial cell apoptosis and increased intestinal permeability after IRI.

TJs between the top of intestinal epithelial cells are responsible for the function of the paracellular barrier and regulate the paracellular transport of substances inside and outside the intestine [30]. Intercellular TJs are multiprotein complexes formed by different junctional molecules, such as occludin and claudins [30]. Additionally, the intracellular scaffold protein zonula occluden-1 (ZO-1) also plays an integral role in TJs [31]. However, there are two different classes of claudins in the intestine: claudin-1, claudin-3, claudin-5, claudin-8, and so on act as sealing claudins, and claudin-2, claudin-10a/-10b, claudin-15, and so on act as pore-forming claudins [32, 33]. According to previous studies, claudin-2 is normally tissue-specifically expressed in the small intestine and overexpressed in leaky epithelial tissues, such as the colonic tissues of inflammatory bowel disease [34, 35]. The results of the present study also showed an increase in claudin-2 expression accompanied by an increase in intestinal permeability after IRI. Importantly, studies have shown that autophagy can induce the degradation of claudin-2, thus enhancing epithelial barrier function [16, 36]. Our data showed that RAP could inhibit the expression of claudin-2 and decrease the permeability of the intestine after intestinal IRI, while 3-MA showed the opposite effects. These results indicated that autophagy induction could protect the structural and functional integrity of the epithelial barrier after intestinal IRI. However, the effects of rapamycin and 3-MA in intestinal epithelial injury after IRI observed cannot be concluded to be merely due to the effects of the compounds on autophagy, as rapamycin is also a mammalian target of rapamycin inhibitor, and 3-MA is a phosphatidylinositol 3-kinase inhibitor; therefore, they will affect cell proliferation, growth, cell cycle, translation, differentiation, etc. Thus, further study is required to prove this point.

In conclusion, the role of the autophagy inducer RAP and the autophagy inhibitor 3-MA in the injury and repair of the epithelial barrier after intestinal IRI was verified. The results showed that upregulation of epithelial cell autophagy by RAP inhibits epithelial cell apoptosis and enhances the function of the intestinal epithelial barrier after acute intestinal IRI. This study suggested that autophagy induction may be a potential protective strategy for the epithelial barrier in a short time after acute intestinal I/R. However, the specific mechanism of autophagy induction and inhibition in intestinal channel dysfunction caused by ischemia–reperfusion is still unclear and must involve a more complex signaling pathway mechanism, which needs to be further explored. At the same time, validation in rats cannot be directly applied to clinical validation. To obtain a more accurate and instructive mechanism of action, corresponding clinical sample experiments are needed in the future.

## Data Availability

The datasets used and/or analyzed during the current study are available from the corresponding author upon reasonable request.
